# Venous Vessel Size Imaging Derived From A Breath‐Hold Task

**DOI:** 10.1002/nbm.70212

**Published:** 2025-12-17

**Authors:** Ke Zhang, Artur Hahn, Simon M. F. Triphan, Mark O. Wielpütz, Christian H. Ziener, Mark E. Ladd, Heinz‐Peter Schlemmer, Hans‐Ulrich Kauczor, Oliver Sedlaczek, Felix T. Kurz

**Affiliations:** ^1^ Department of Diagnostic and Interventional Radiology Heidelberg University Hospital Heidelberg Germany; ^2^ Translational Lung Research Center (TLRC), German Center for Lung Research (DZL) Heidelberg Germany; ^3^ Department of Diagnostic and Interventional Radiology with Nuclear Medicine Thoraxklinik at Heidelberg University Hospital Heidelberg Germany; ^4^ Division of Radiology German Cancer Research Center Heidelberg Germany; ^5^ Rupercht‐Karls University Heidelberg Heidelberg Germany; ^6^ Division of Medical Physics in Radiology German Cancer Research Center Heidelberg Germany; ^7^ Faculty of Physics and Astronomy Heidelberg University Heidelberg Germany; ^8^ Faculty of Medicine Heidelberg University Heidelberg Germany; ^9^ Division of Neuroradiology Geneva University Hospitals Geneva Switzerland

**Keywords:** BOLD, breath‐hold task, vessel size imaging

## Abstract

Vessel size imaging (VSI) to provide a measure of vessel radius in the brain has been demonstrated using an injection of contrast agent. Venous vessel radius imaging of brain microvasculature is also possible by exploiting hypercapnia and hyperoxia. However, these respiratory challenges need external devices like masks, monitors, and gas application. In this study, we employ a breath‐hold task to mimic hypercapnia for venous VSI. Breath‐hold experiments of brain scans were performed on 14 subjects on a 3‐T scanner. Parametric maps of mean venous vessel radius were calculated from the changes in R2* and R2, ΔR2* and ΔR2, respectively, which were measured by simultaneous acquisition of gradient‐echo and spin‐echo signals using a spin‐ and gradient‐echo (SAGE) echo‐planar imaging sequence. In addition, we numerically simulated the expected transverse relaxation in voxels with different vessel radii based on randomly distributed cylinders to eventually obtain vessel size index q (ΔR2*/ΔR2) and associated average vessel radii. With this empirical relation of vessel size index q and vessel radius, the mean measured vessel size index was determined, and venous vessel radii in breath–hold were found to be 7.18 ± 0.49 μm in gray matter and 6.06 ± 0.22 μm in white matter. This study demonstrates the feasibility of venous VSI using a simple breath–hold task. The approach avoids contrast agents and specialized gas delivery, providing a practical alternative for assessing vascular properties. Our results show good agreement with previous hypercapnia– and contrast–based studies, supporting the validity of this noninvasive method.

## Graphical abstract

In this study, we propose the utilization of a spin‐ and gradient‐echo echo‐planar imaging approach, in conjunction with a numerical simulation, for the quantitative assessment of venous vessel size measurements during a breath‐hold task.

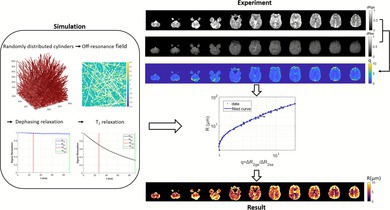

**Abbreviations**
BH: breath‐hold
BOLD: blood oxygen level–dependent
CBV: cerebral blood volume
EPI: echo‐planar imaging
FB: free breathing
FoV: field of view
GE/SE: gradient‐echo/spin‐echo
GM: gray matter
Hct: hematocrit
iPAT: integrated parallel acquisition techniques
MPRAGE: magnetization prepared rapid gradient‐echo imaging
SAGE: spin‐ and gradient‐echo
SNR: signal‐noise ratio
TE: echo time
TR: repetition time
VSI: vessel size imaging
WM: white matter

## 1 Introduction

Vessel size imaging (VSI) with MRI is a technique for the noninvasive measurement of parameters that describe the structural heterogeneity of brain microvasculature [1, 2, 3, 4]. VSI was first proposed by Tropres et al. [5] by injection of a paramagnetic contrast agent to explore the intrinsic contrast difference between gradient‐echo (GE) and spin‐echo (SE) signals, which exhibit distinct dependencies on the blood vessel radius: SE is more sensitive to microvascular susceptibility changes, whereas GE is sensitive to both macrovascular and microvascular susceptibility changes [6]. The GE and SE signal changes, in turn, depend on the respective changes in the transverse relaxation rates, Δ*R*_2_* and Δ*R*_2_. The ratio *q =* Δ*R*_2_/Δ*R*_2_*, also referred to as the vessel size index, can be expressed in terms of the mean vessel radius *R* within a voxel [5, 7, 8, 9, 10, 11, 12]. VSI using a contrast agent has been applied in brain tumor [13] and stroke studies [14]. VSI has the potential to assess the angiogenic activity of brain tumors and to evaluate the effect of antiangiogenic therapy [13]. It also reveals sex‐specific changes in cerebral microvasculature with aging [2].

In addition to the injection of a contrast agent, changes in the transverse relaxation rates *R*_2_* and *R*_2_ can also be induced by altering the blood oxygenation level–dependent (BOLD) contrast through hypoxia, hypercapnia, or hyperoxia. Hypercapnia and hyperoxia give rise to vasodilation and vasoconstriction, respectively [15, 16]. The influence of hypercapnia and hyperoxia on venous vessel size in the human brain has been studied [7, 9, 11, 17], where venous vessel radii could be measured in response to both conditions [7]. The venous vessel radii could be determined by calculation of the changes in *R*_2_* and *R*_2_ that are induced by breathing 6% CO_2_ or pure oxygen [7]. This alternative approach based on BOLD effects is particularly attractive since it does not require intravenous contrast agents. However, these respiratory challenges also need external devices such as special masks and monitors; additional preparations in gas supply and oxygen may not be supplemented to specific patient populations [18].

In this work, we examine VSI based on a breath‐hold (BH) task at room air to mimic externally induced hypercapnia. Parametric maps of mean venous vessel radius were calculated from Δ*R*_2_* and Δ*R*_2_, which were measured by simultaneous acquisition of GE and SE signals. To do so, we have numerically simulated the expected transverse relaxation in NMR voxels with different vessel radii based on randomly distributed cylinders to determine the relation of *q* and vessel radius. After applying this relation to the measured *q*, venous vessel radius *R* was calculated.

## 2 Methods

### 2.1 Measurement Setup

A total of 14 healthy volunteers (5 female, 9 male, aged 30 ± 5 years) were examined prospectively using a 20‐channel head coil on a 3‐T MRI scanner (Magnetom Prisma, Siemens Healthineers, Erlangen, Germany). All participants provided written informed consent, and the study was approved by the institutional ethics committee.

To measure VSI, a BH respiratory challenge was integrated into an existing brain imaging protocol: for block‐designed BH tasks (Supplementary Figure S1a), 110 measurements were obtained, which included five and a half BH/FB (free breathing) cycles with 20 measurements (34 s) per cycle and 10 measurements (17 s) per half cycle. BH was performed after inspiration. A spin‐ and gradient‐echo (SAGE) EPI sequence was developed to capture both GE and SE signals [19]. Specific sequence parameters were as follows: FOV = 220 × 220 mm^2^, matrix size = 64 × 64 × 28, resolution = 3.4 × 3.4 × 3.5 mm^3^, slice gap = 0.7 mm, in‐plane iPAT factor = 2, multiband factor = 2, bandwidth = 1776 Hz/px, TE_GE_/TE_SE_/TR = 27.08/90/1700 ms. Assuming a monoexponential signal decay, Δ*R*_2_* and Δ*R*_2_ were calculated according to [7]:

(1) $$\begin{array}{c} ∆R_{2}^{*}=\frac{-\ln\left(S_{\mathrm{GE}}\left(\mathrm{BH}\right)/S_{\mathrm{GE}}\left(\mathrm{FB}\right)\right)}{{\mathrm{TE}}_{\mathrm{GE}}}\\ ∆R_{2}=\frac{-\ln\left(S_{\mathrm{SE}}\left(\mathrm{BH}\right)/S_{\mathrm{SE}}\left(\mathrm{FB}\right)\right)}{{\mathrm{TE}}_{\mathrm{SE}}}, \end{array}$$

where *S*_GE_ (BH), *S*_GE_ (FB), *S*_SE_ (BH), and *S*_SE_ (FB), are the averaged GE and SE signal intensities during BH and FB, respectively. Finally, the vessel size index was calculated according to:

(2) $$q=\frac{∆R_{2}^{*}}{∆R_{2}}.$$

### 2.2 Simulations

**FIGURE 1:**
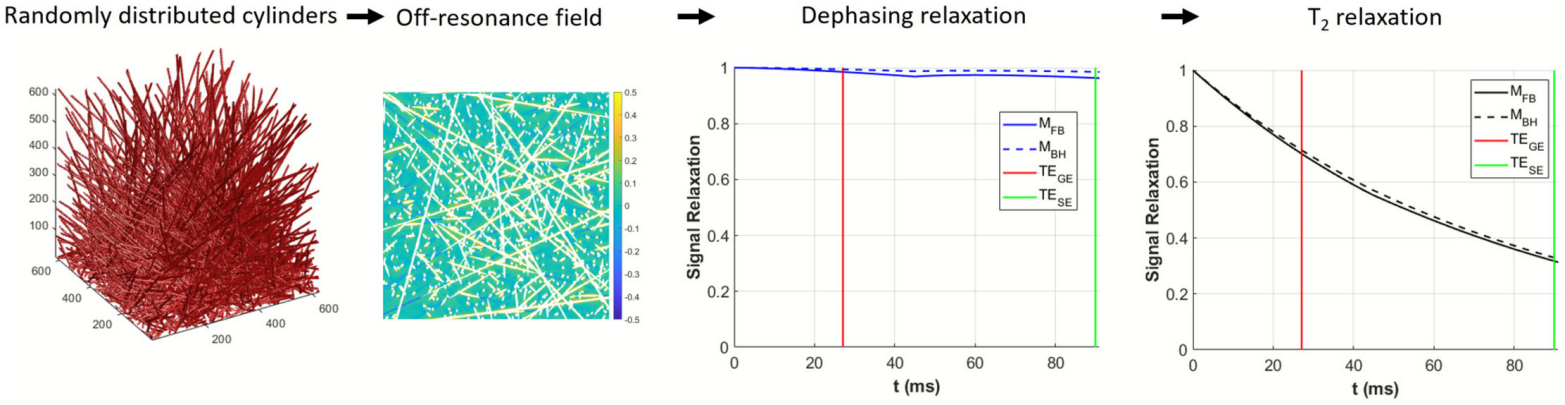
Simulation processing. Schematic flowchart of the numerical processing conducted on randomly distributed cylinders. Following the determination of the blood vessel–induced off‐resonance frequency distribution within the virtual voxel, the extravascular water proton signal was numerically simulated in FID conditions. The magnetization decay is accountable to T_2_ decay.

To find the relationship between vessel radii *R* and vessel size index *q*, a numerical simulation was performed [5]. As illustrated in Figure 1, randomly oriented infinite cylinders with different vessel radii *R* were defined with a blood volume fraction of 4% [20]. Simulations were based on 49 different *R* distributed exponentially within the range of 0.4–100 μm. The off‐resonance frequency Δ*ω*(*r*) was calculated based on an estimated average blood oxygenation of *ϒ* = 0.6 for FB and 0.75 for BH [7, 21], with hematocrit (Hct) = 0.4 and field strength *B*_0_ = 3 T. The phase evolution of isochromates placed randomly throughout the tissue was simulated in the rotating frame of *ω*_0_ *= γB*_0_, based on the local off‐resonance frequencies *Δω*(*r*) encountered during a 3D discrete‐time, continuous‐space random walk with tunable time steps and Gaussian distributed step size to model water diffusion [22]. This random walk implementation was motivated by the Bloch–Torrey equation, which governs the NMR signal evolution with diffusion effects. In our simulations, the vessels acted as impermeable diffusion barriers toward the isochromates and we omitted longitudinal relaxation with T_1_. Further simulation parameters were as follows: virtual voxel size 500^3^ μm^3^; mean extravascular spin packet density of 5 μm^−3^; water proton diffusion coefficient *D* = 1 μm^2^/ms; diffusion time step *δt* = 0.1 ms; T_2,tissue_ = 83.5 ms; T_2,blood_ (FB) = 32.3 ms; T_2,blood_ (BH) = 53.2 ms as described in the literature [7]; resolution of grid with calculated field distortion: 0.8 × 0.8 × 0.8 μm^3^. For this simulation, we used a custom‐developed pipeline written in C++11 with OpenMP multithreading version 3.1 (OpenMP Architecture Review Board, https://www.openmp.org/) and MATLAB R2020a (Mathworks, Natick, MA) [22].

### 2.3 Data Analyses

All acquired images were processed and analyzed using MATLAB and SPM12 (Wellcome Trust Centre for NeuroImaging, UK). GE and SE EPI images were realigned and resliced to remove the variance of head motion. Slice time correction was applied subsequently. A 5‐mm full‐width at half‐maximum isotropic Gaussian spatial smoothing kernel was applied to all EPI volumes. First‐level analysis was performed using a general linear model (GLM) that included the canonical hemodynamic response function (HRF) and its temporal derivative, along with nuisance regressors (motion parameters). A *t*‐test was used to identify statistically significant signal changes between periods of BH and FB. Only pixels that showed significant signal increases in both the GE and the SE data were included (*p* < 0.001). The predicted signal was derived from the design matrix by combining the beta estimates of the HRF and temporal derivative. Circular shifting was applied to the time series to compensate for hemodynamic lag. Signals of BH and FB were averaged based on the shifted predicted signal and used to calculate Δ*R*_2_* and Δ*R*_2_ (Equation 1). Furthermore, *q* was derived (Equation 2) and fitted into simulated results to calculate VSI. Finally, calculated VSI was normalized to Montreal Neurological Institute (MNI) standard space. To statistically compare VSI values between GM and WM within subjects, the nonparametric Wilcoxon signed‐rank test was applied. To test reproducibility, we performed a test–retest experiment in three subjects who each completed the identical BH paradigm twice (separate sessions; Supplementary Figure S1).

## 3 Results

**FIGURE 2:**
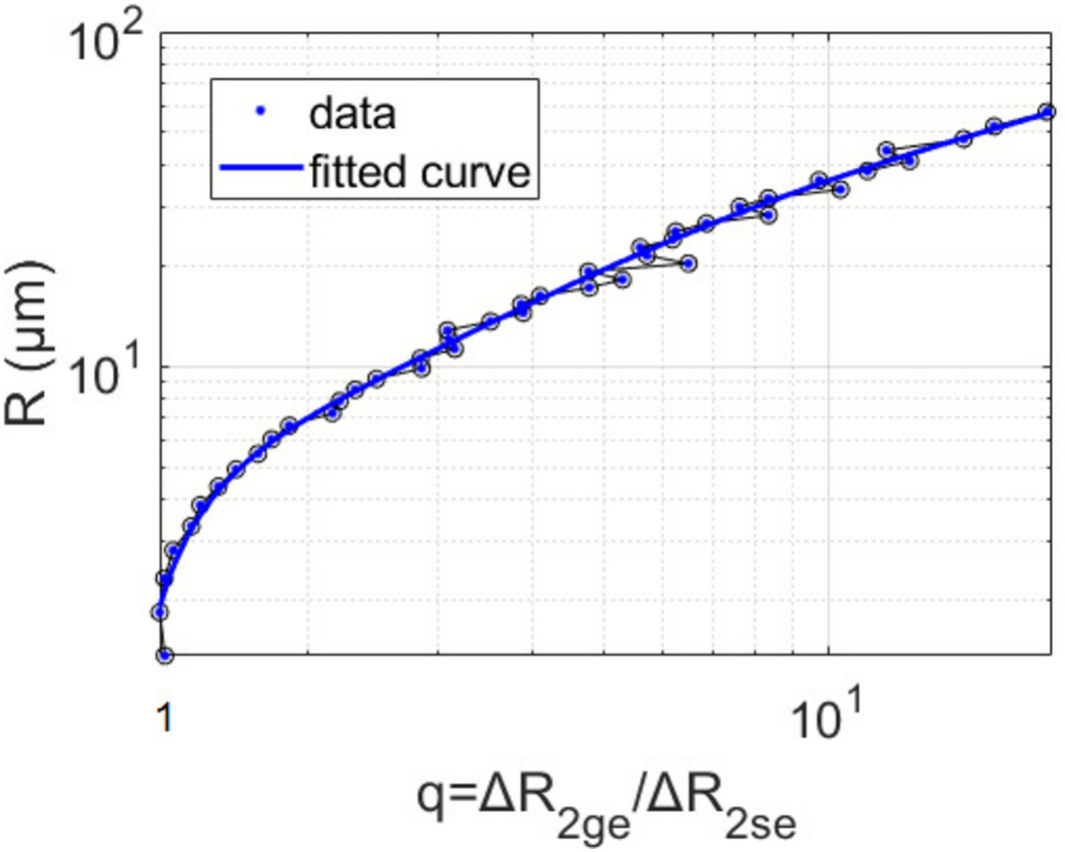
Dependence of the mean vessel radius *R* on the vessel size index *q* for BH. A sixth‐order logarithmic was fitted on this dependency. Please note the double‐logarithmic scale.

The dependence of mean vessel radius *R* on the vessel size index *q* found using the simulation is shown in Figure 2. To achieve a higher adjusted *R* ^*2*^ a calibration curve was constructed by fitting a sixth‐order logarithmic equation:

(3) $$R\left(q\right)=\sum_{n=0}^{6}P_{n}\ln^{n}\left(q\right).$$

The fit was performed by nonlinear least squares implemented in MATLAB. Robust regression residuals using the bisquare weight function were applied. *P*_*n*_ was fitted as [−2.70 24.82 −27.26 20.49 −5.89 0.64].

**FIGURE 3:**
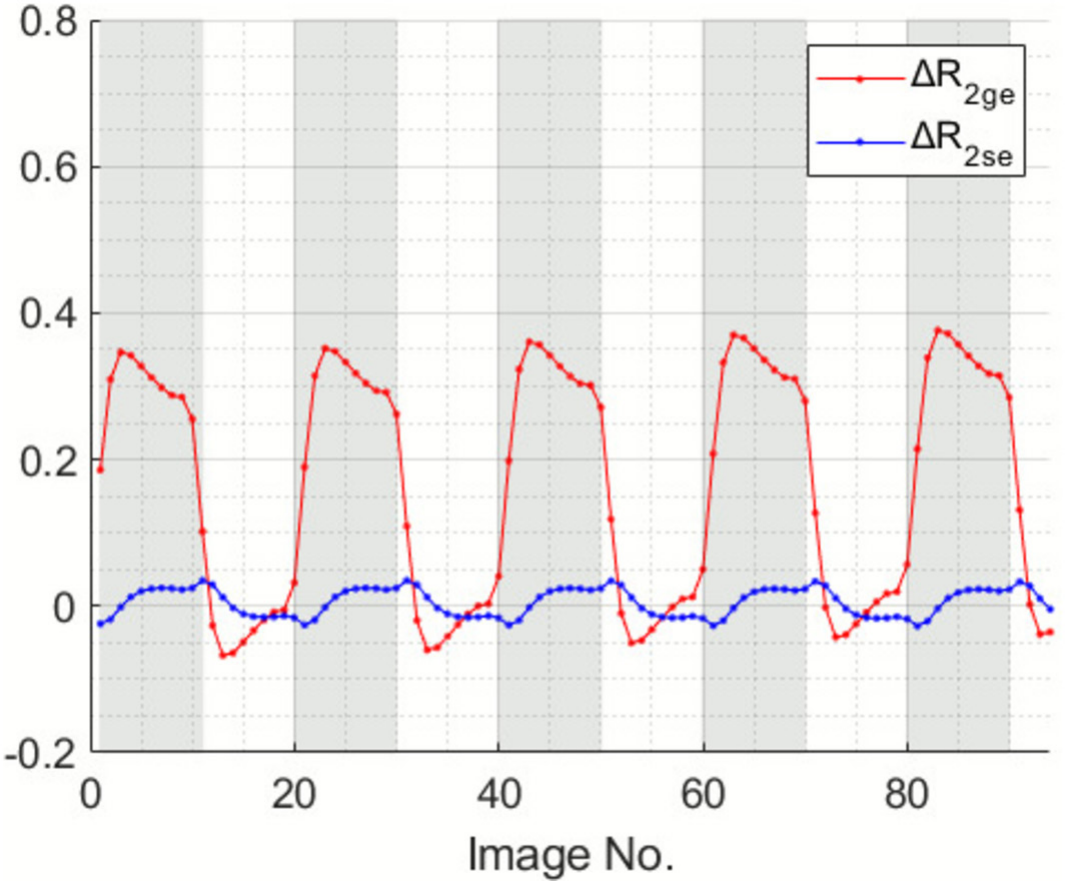
Predicated GE and SE signal changes in a selected pixel of a representative subject in response to BH tasks. Gray region indicates the time intervals during BH.

**FIGURE 4:**
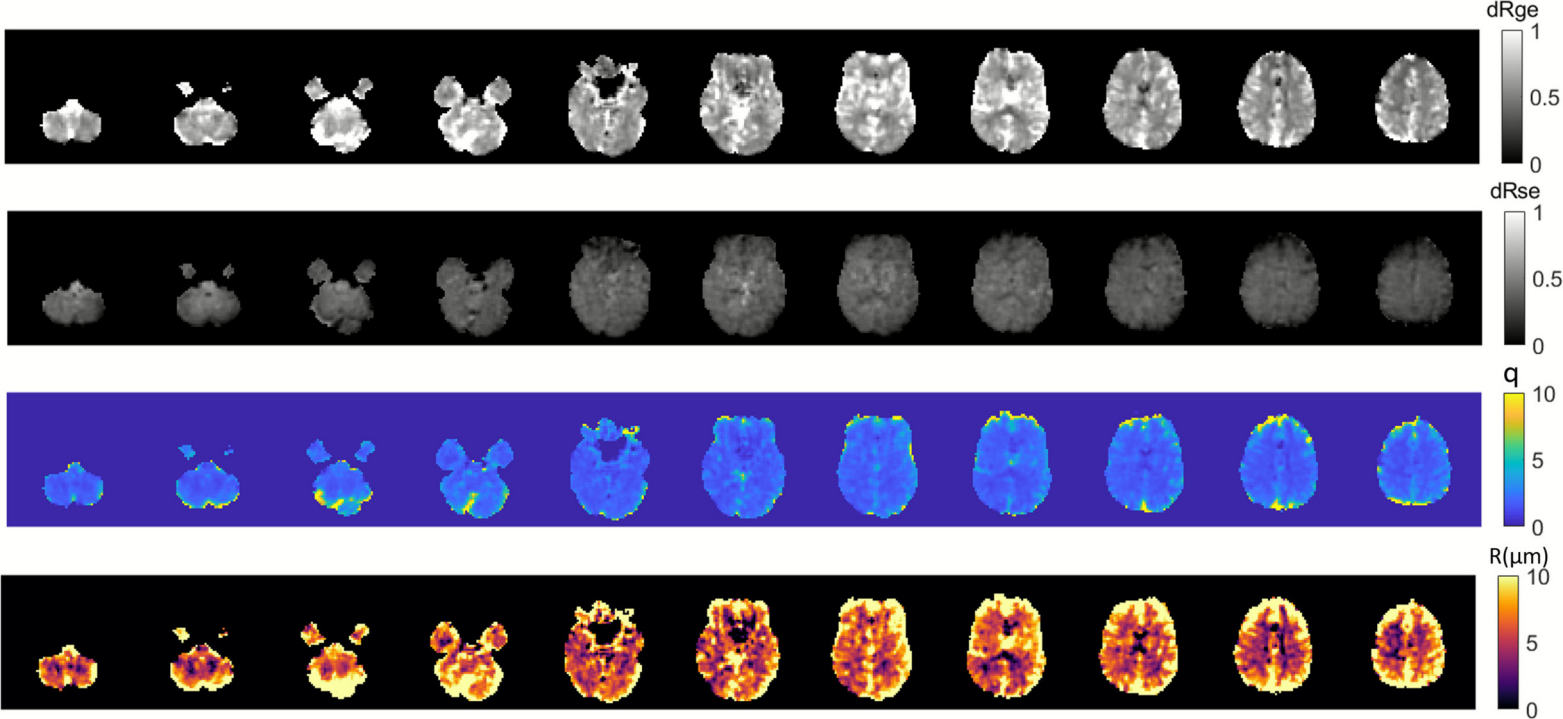
The calculated Δ*R*_2_^*^ (dRge) and Δ*R*_2_ (dRse) maps in response to BH. With *q =* Δ*R*_2_*/Δ*R*_2_, VSI is calculated based on *q* and fitting results.

Representative calculated Δ*R*_2_* and Δ*R*_2_ curves based on predicted GE and SE signal changes during the BH tasks are shown in Figure 3. These two signals were selected at the same position in the representative images. Figure 4 shows the calculated Δ*R*_2_* (dRge) and Δ*R*_2_ (dRse) maps in response to BH. The parameter *q* represents the ratio between them, and VSI in micrometers was calculated based on *q* and the fitting equation. GE is sensitive to both macrovascular and microvascular susceptibility changes. Especially, Δ*R*_2_* showed higher signal at the large draining veins. SE is sensitive to microvascular susceptibility changes, and accordingly, Δ*R*_2_ showed less signal at the big vessels. After dividing Δ*R*_2_* by Δ*R*_2_, we would expect to see most changes in the big vessels such as regions at the sagittal superior sinus and around the pentagon cistern.

**FIGURE 5:**
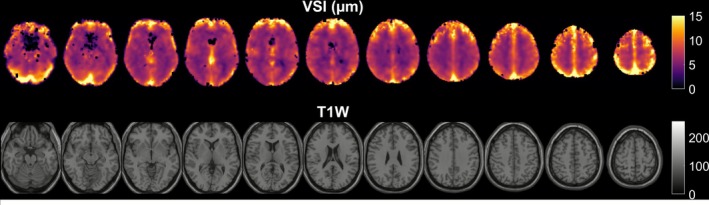
Mean VSI (top) averaged from 14 subjects in MNI standard space compared to T_1_ weighted (down) in the same space.

Figure 5 shows the averaged VSI from all subjects in MNI standard space. The VSI in the big vessels showed higher values, as expected. VSI was largest in the sulci, where we expect the leptomeningeal vessels, as well as around the supply area of the middle cerebral artery (temporoinsular region) and basal ganglia.

**FIGURE 6:**
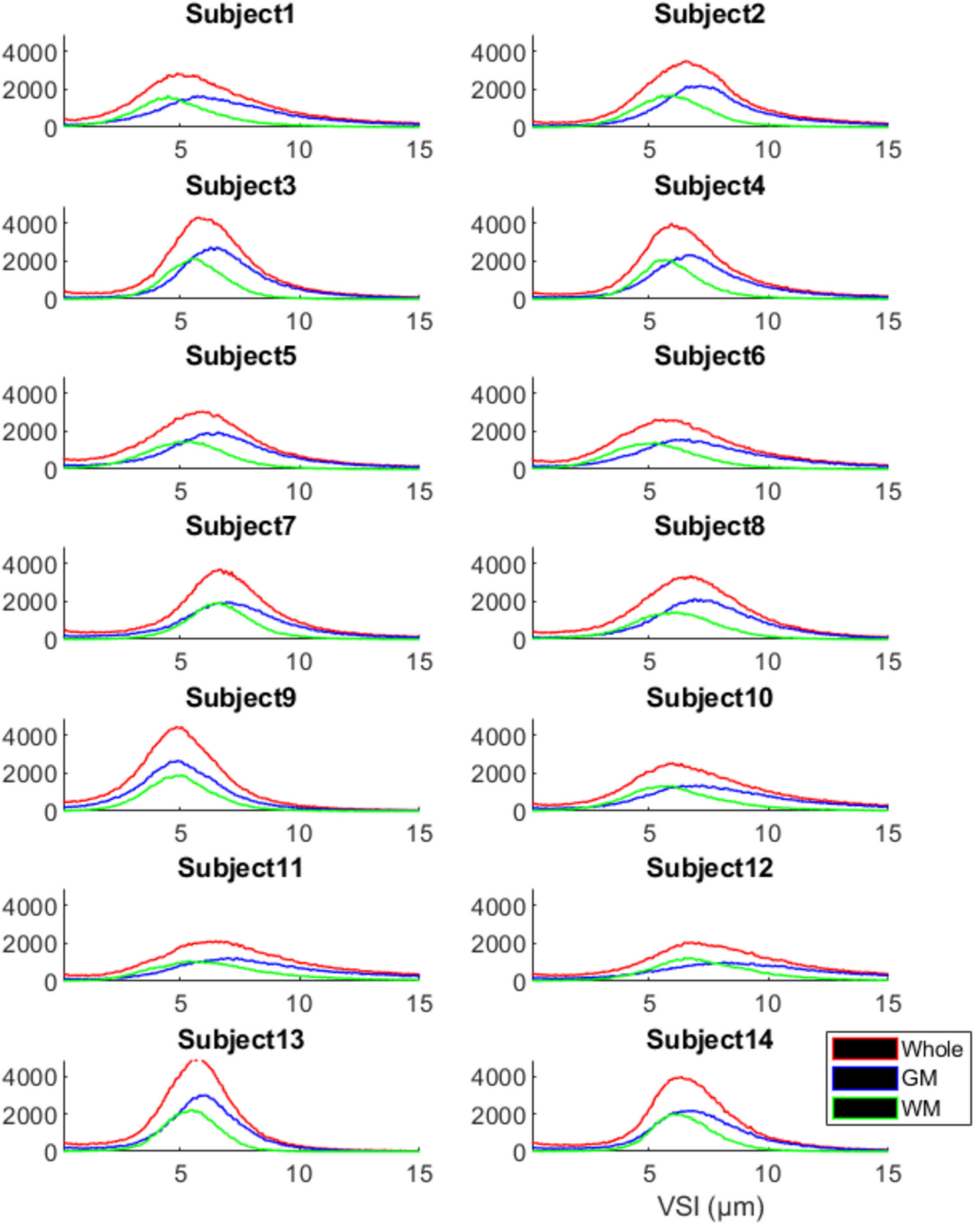
Distribution of vessel radius from individual subjects. Red lines are the histograms of vessel radius in whole brain. Blue lines are the ones in gray matter (GM) and green lines are the ones in white matter (WM).

Figure 6 shows the plots of distributions of venous vessel radii in the whole brain, GM, and WM from all 14 subjects.

**FIGURE 7:**
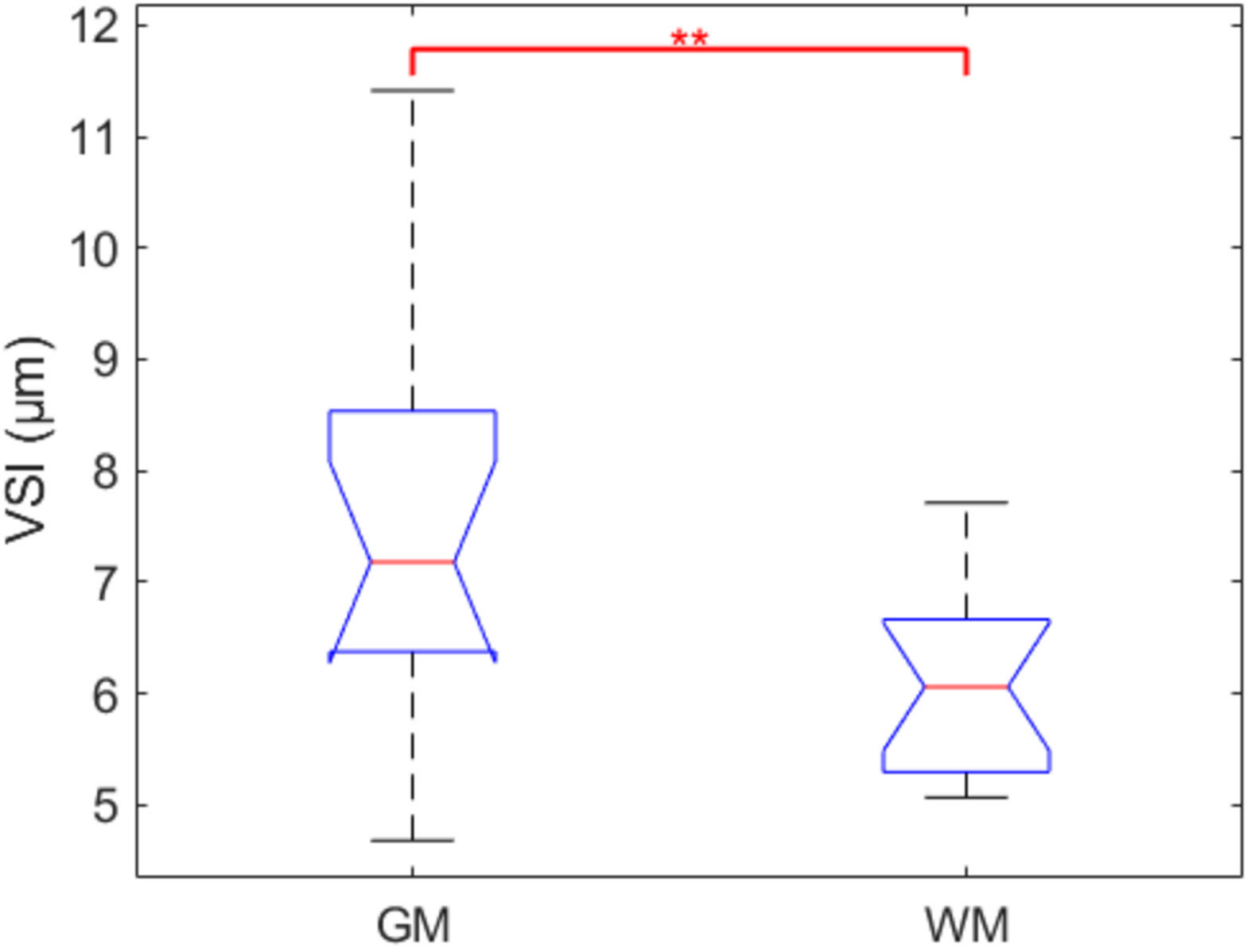
Comparison of VSI in GM and WM across 14 subjects. The differences between them were significant (*p* < 0.01).

The mean venous vessel radii in gray matter (GM) and white matter (WM) in each individual subject are compared in Figure 7. The difference between them is significant (*p* < 0.01). Mean venous vessel radii in GM and WM were 7.18 ± 0.49 and 6.06 ± 0.22 μm, respectively.

Mean intraclass correlation coefficients (ICC) were calculated at five lobes of BH‐VSI across three tests as 0.346 ± 0.304 and across two paradigms at the same day as 0.35 ± 0.063 (Supplementary Table S1).

## 4 Discussion

In this study, a SAGE sequence was employed to measure GE and SE signals for the assessment of changes in *R*_2_* and *R*_2_ induced using the BH task. We have numerically simulated the expected transverse relaxation in NMR voxels with different vessel radii based on randomly distributed cylinders.

The relation of vessel size index *q* = (Δ*R*_2_*/Δ*R*_2_) and vessel radius was then determined based on this simulation. After applying this relation to the measured *q*, venous vessel radius *R* could be calculated. The mean venous vessel radii obtained in this study were 7.18 ± 0.49 μm in GM and 6.06 ± 0.22 μm in WM. In a previous study, the mean venous vessel radii during hypercapnia were 7.3 ± 0.3 μm in GM and 6.6 ± 0.5 μm in WM, respectively [7]. Our results were very close to these parameters. While our estimates gave larger radii, estimates in GM did agree quite well with previous results in GM published by Jochimsen et al. [9] of 13.4 μm and Germuska and Bulte [17] of 13.5 μm, suggesting that Shen et al. [7] may have underestimated the radii. Compared with WM, previous VSI studies with contrast agent found cortical GM to have larger mean vascular calibers (5.80 ± 0.59 versus 4.25 ± 0.62, *p* < 0.001) [2, 23]. These values are lower than our current and previous studies [7]. The higher values in our study and in other noncontrast VSI approaches [7, 9, 17] may reflect methodological differences in vascular modeling or the sensitivity of BH and hypercapnia paradigms.

Regarding the reproducibility, we added a test–retest experiment in three subjects (Supplementary Figure S1–5) who each completed the identical BH paradigm twice (separate sessions, Supplementary Figure S1). We now report region‐wise reproducibility of the derived vessel radius using ICC (Supplementary Table S1). These experiments show some consistent estimates across sessions on the same day, supporting the precision of the current BH‐VSI paradigm. However, reproducibility across sessions conducted on different days was lower, suggesting that the BH‐VSI may be influenced by physiological variability between days.

An overestimation of VSI in the frontal lobe was observed (Figure 5). This effect is likely explained by residual artifacts associated with both EPI susceptibility distortions and head motion induced by the BH task. Although nuisance regressors were incorporated into the GLM to account for motion‐related variance, such corrections may not fully eliminate nonlinear or transient motion effects, particularly in regions near air–tissue interfaces where EPI distortion is most pronounced. Future work could address these limitations through the application of field‐map [24] or topup‐based distortion correction [25, 26], more advanced motion modeling (e.g., volume censoring [27] or ICA‐based denoising [28]), or the use of acquisition strategies less sensitive to susceptibility artifacts, such as multiecho EPI [29]. Incorporating these refinements may improve the reliability of VSI estimates in frontal regions and enhance the overall robustness of BH‐based approaches.

Our method differs from traditional gas challenge approaches, notably by avoiding the need for specialized gas delivery systems and the use of contrast agents, thereby making it more accessible for certain patient populations. Although this approach may produce different spatial patterns [7], preliminary validation studies have demonstrated promising correlations with established anatomical and physiological data [9, 17].

There are multiple limitations to this study. First, cerebral blood volume (CBV) used for the simulation was set to 4%. However, it was found that the vessel size index *q* is relatively insensitive to variable blood volume fractions [7]. Second, blood oxygenation was assumed at *ϒ* ≈ 0.6 for FB and 0.75 for BH. T_2_ and T_2_* values of the blood and tissue were also based on literature in the simulation. These parameters might be different for different subjects. A multiecho SAGE sequence [29] for quantitative measurements of T_2_ and T_2_* could be used to quantify explicit oxygenation levels and the relaxation times used in numerical simulations. Third, the BH task with 17 s BH duration provides less BOLD signal‐noise ratio (SNR) compared to more pronounced hypercapnia and hypoxia approaches. However, the BH task also has less impact on the induced change of vessel size compared to hypercapnia and hypoxia approaches: hypercapnia causes vasodilation and hyperoxia induces vasoconstriction. It should be noted that BH‐induced BOLD responses are generally smaller than those elicited by gas challenges, which could make the estimated vessel radii more sensitive to noise and variability in BH compliance. While our simulations encompass the range of signal amplitudes observed in our data, future work could further explore these effects using dedicated simulations or alternative experimental designs. Finally, this study lacks an in vivo gas‐based benchmark to directly validate the estimated vessel radii. Gas challenges have been widely used in prior studies to establish the accuracy of vessel size modeling approaches. However, such experiments were not performed here due to logistical and ethical constraints associated with gas delivery. Consequently, the validation of our results relies primarily on simulations and consistency with prior literature. Future studies incorporating gas‐based experiments would be valuable to further verify and refine the methodology.

## 5 Conclusion

In conclusion, this study demonstrates the feasibility of estimating venous VSI using a simple BH task. By eliminating the need for specialized gas delivery systems or contrast agents, this approach offers a practical and accessible alternative for assessing vascular properties in both research and clinical settings. The close agreement of our results with previous hypercapnia‐ and contrast‐based VSI studies supports the validity of this method, while observed differences highlight the importance of continued methodological refinement. Future work with larger cohorts, improved motion and distortion correction, and direct comparisons with gold‐standard techniques will be essential to further establish the accuracy and translational potential of BH‐based VSI.

## Author Contributions

**Ke Zhang:** funding, conceptualization, MRI pulse sequence development, data acquisition, formal MRI data analysis, investigation, data curation, visualization, writing – original draft, visualization, and editing. **Artur Hahn:** simulation, writing, review, and editing. **Simon M. F. Triphan:** data acquisition, writing, review, and editing. **Mark O. Wielpütz:** funding, writing, review, and editing. **Christian H. Ziener:** writing, review, and editing. **Mark E. Ladd:** resources, writing, review, and editing. **Heinz‐Peter Schlemmer:** resources, writing, review, and editing. **Hans‐Ulrich Kauczor:** resources, writing, review, and editing. **Oliver Sedlaczek:** writing, review, and editing. **Felix T. Kurz:** resources, supervision, funding, project administration, writing, review, and editing.

## Funding

This work was supported by the Deutsche Forschungsgemeinschaft (DFG, German Research Foundation), 507778062. This study was supported by grants from the Bundesministerium für Bildung und Forschung (BMBF, German Federal Ministry of Education and Research) 82DZL004A1.

## Conflicts of Interest

The authors declare no conflicts of interest.

## Supporting information


**Figure S1:** Two BH paradigms were tested for reproducibility: (a) an experimental design for the current study and one for comparison (b). In the current design (a), 110 measurements were obtained, which included five and a half BH (breath‐holding)/FB (free breathing) cycles with 20 measurements (34 s) per cycle and 10 measurements (17 s) per half cycle. For the comparison (b), 160 measurements were obtained, which included five FB cycles with 30 measurements (51 s) per cycle and 10 measurements (17 s) per BH.
**Figure S2:** Predicated time series of GE and SE in the same pixel of a representative subject in response to two different tasks. Gray region indicates the time intervals during BH.
**Figure S3:** Calculated and spatially normalized vessel radius of the first representative subject (male, 40 years) after task (a) in day 1 (R1), after task (a) in day 2 (R2), and after task (b) in day 2 (R3).
**Figure S4:** Calculated and spatially normalized vessel radius of the second representative subject (male, 28 years) after task (a) in day 1 (R1), after task (a) in day 2 (R2) and after task (b) in day 2 (R3).
**Figure S5:** Calculated and spatially normalized vessel radius of the third representative subject (male, 28 years) after task (a) in day 1 (R1), after task (a) in day 2 (R2) and after task (b) in day 2 (R3).
**Table S1:** Intraclass correlation coefficients (ICC) were calculated at five lobes of BH‐VSI across three tests (R1–3) and across two paradigms at the same day (R2–3).

## Data Availability

The data that support the findings of this study are available from the corresponding author upon reasonable request.
