# Supplementary material for: Venous Vessel Size Imaging Derived From A Breath‐Hold Task
Source: NMR Biomed. 2025 Dec 17;39(1):e70212. doi: 10.1002/nbm.70212 (PMC12710682; doi:10.1002/nbm.70212)
Supplement: Supplementary file 1 — Figure S1: Two BH paradigms were tested for reproducibility: (a) an experimental design for the current study and one for comparison (b). In the current design (a), 110 measurements were obtained, which included five and a half BH (breath‐holding)/FB (free breathing) cycles with 20 measurements (34 s) per cycle and 10 measurements (17 s) per half cycle. For the comparison (b), 160 measurements were obtained, which included five FB cycles with 30 measurements (51 s) per cycle and 10 measurements (17 s) per BH. Figure S2: Predicated time series of GE and SE in the same pixel of a representative subject in response to two different tasks. Gray region indicates the time intervals during BH. Figure S3: Calculated and spatially normalized vessel radius of the first representative subject (male, 40 years) after task (a) in day 1 (R1), after task (a) in day 2 (R2), and after task (b) in day 2 (R3). Figure S4: Calculated and spatially normalized vessel radius of the second representative subject (male, 28 years) after task (a) in day 1 (R1), after task (a) in day 2 (R2) and after task (b) in day 2 (R3). Figure S5: Calculated and spatially normalized vessel radius of the third representative subject (male, 28 years) after task (a) in day 1 (R1), after task (a) in day 2 (R2) and after task (b) in day 2 (R3). Table S1: Intraclass correlation coefficients (ICC) were calculated at five lobes of BH‐VSI across three tests (R1–3) and across two paradigms at the same day (R2–3). [file NBM-39-e70212-s001.docx]

Supplementary file

Figure S1. Two BH-paradigm were tested for reproducibility: (a) experiment design for the current study and for a comparison (b). As current design (a) 110 measurements were obtained, which included five and a half BH (breath holding)/FB (free breathing) cycles with 20 measurements (34s) per cycle and 10 measurements (17s) per half cycle. For comparison (b), 160 measurements were obtained, which included five and a FB cycles with 30 measurements (51s) per cycle and 10 measurements (17s) per BH.


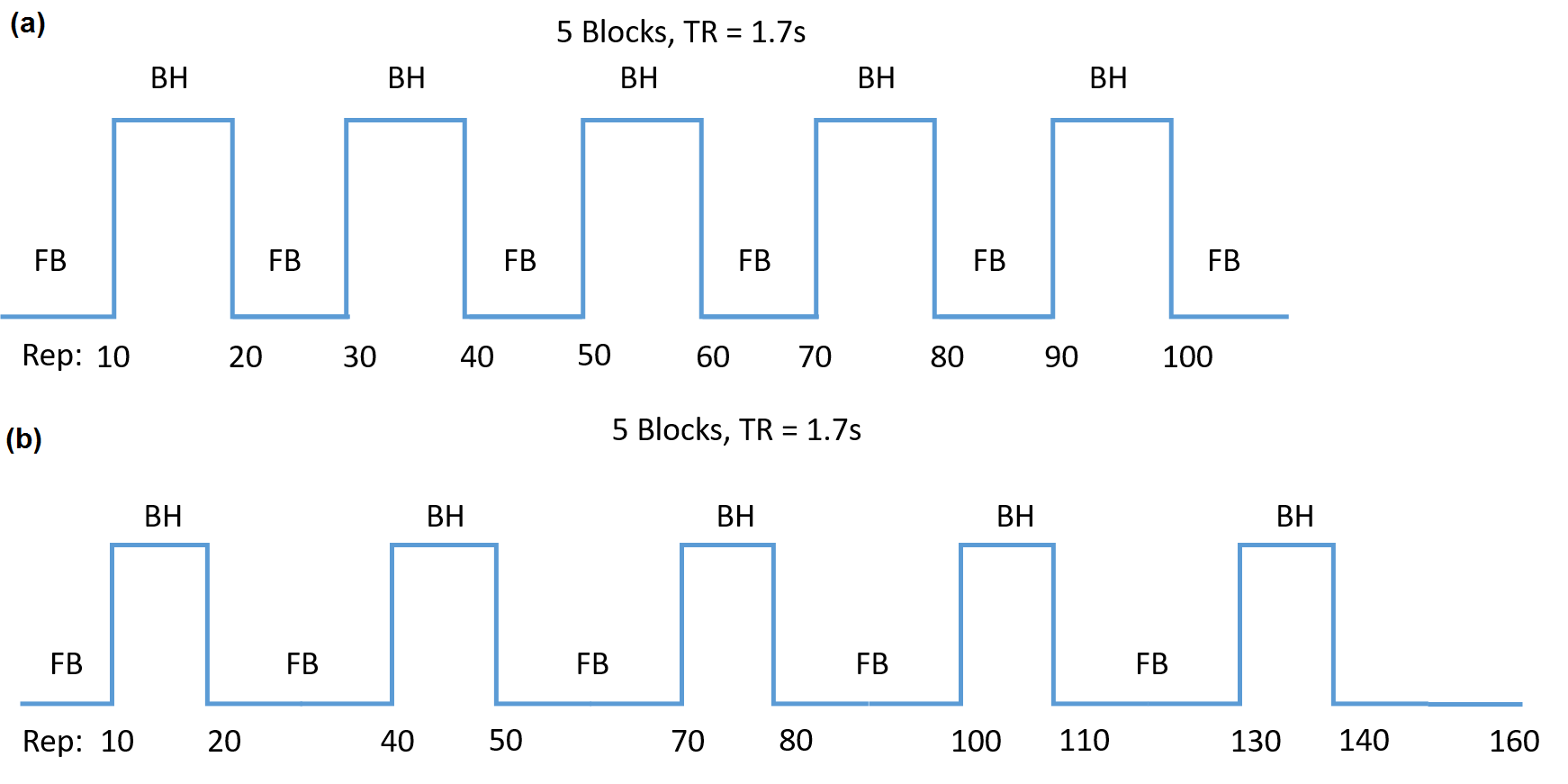


Figure S2. Predicated time series of GE and SE in a same pixel of a represent subject in response to two different tasks. Gray region indicate the time intervals during breath holding:


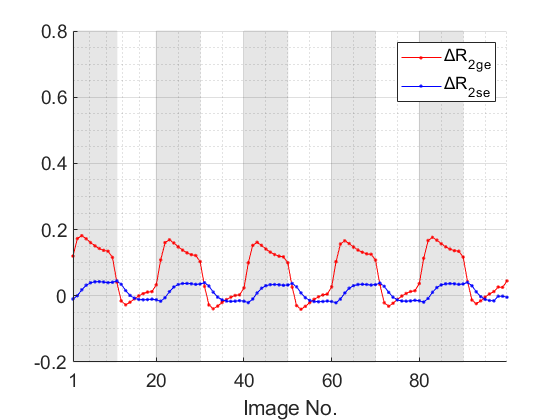


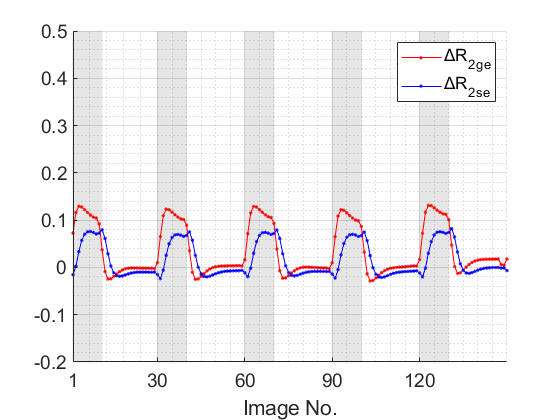


Figure S3. Calculated and spatially normalized vessel radius of the first represent subject (male, 40 years) after task (a) in day 1 with the title of R1, after task (a) in day 2 with the title of R2 and after task (b) in day2 with the title of R3.


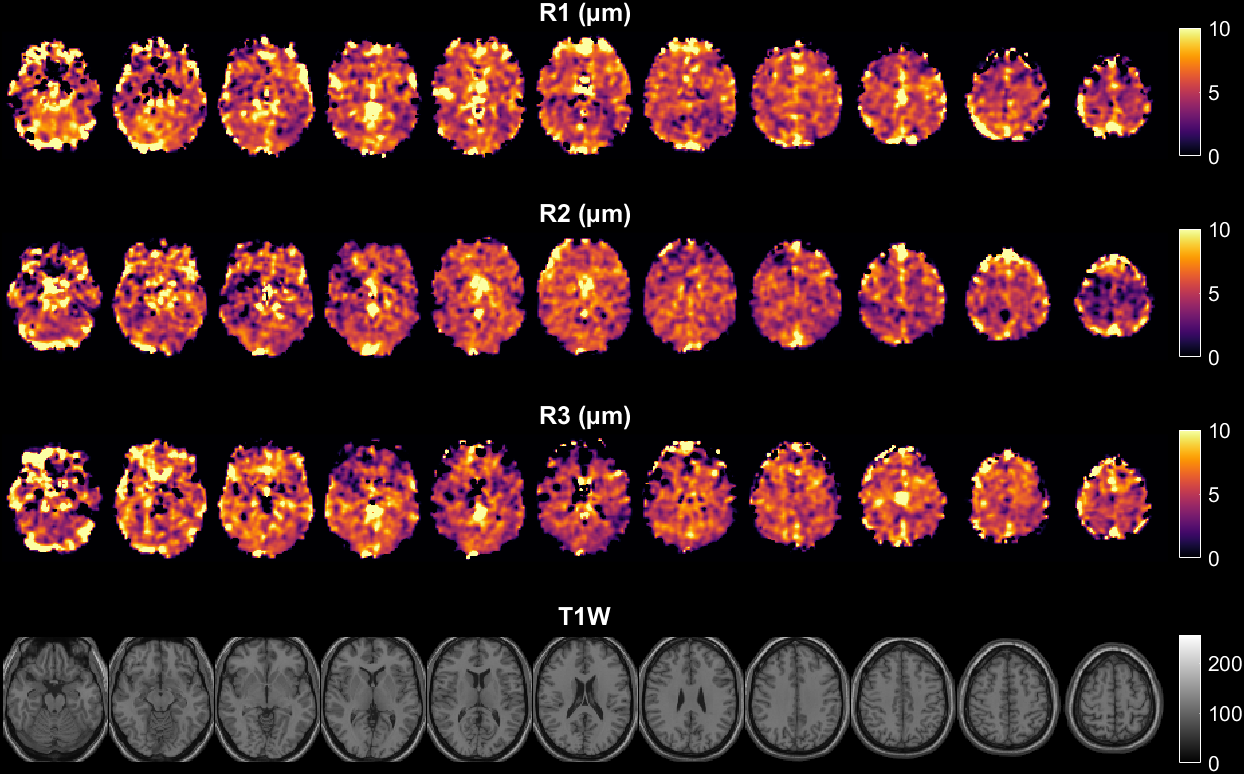


Figure S4. Calculated and spatially normalized vessel radius of the second represent subject (male, 28 years) after task (a) in day 1 with the title of R1, after task (a) in day 2 with the title of R2 and after task (b) in day2 with the title of R3.


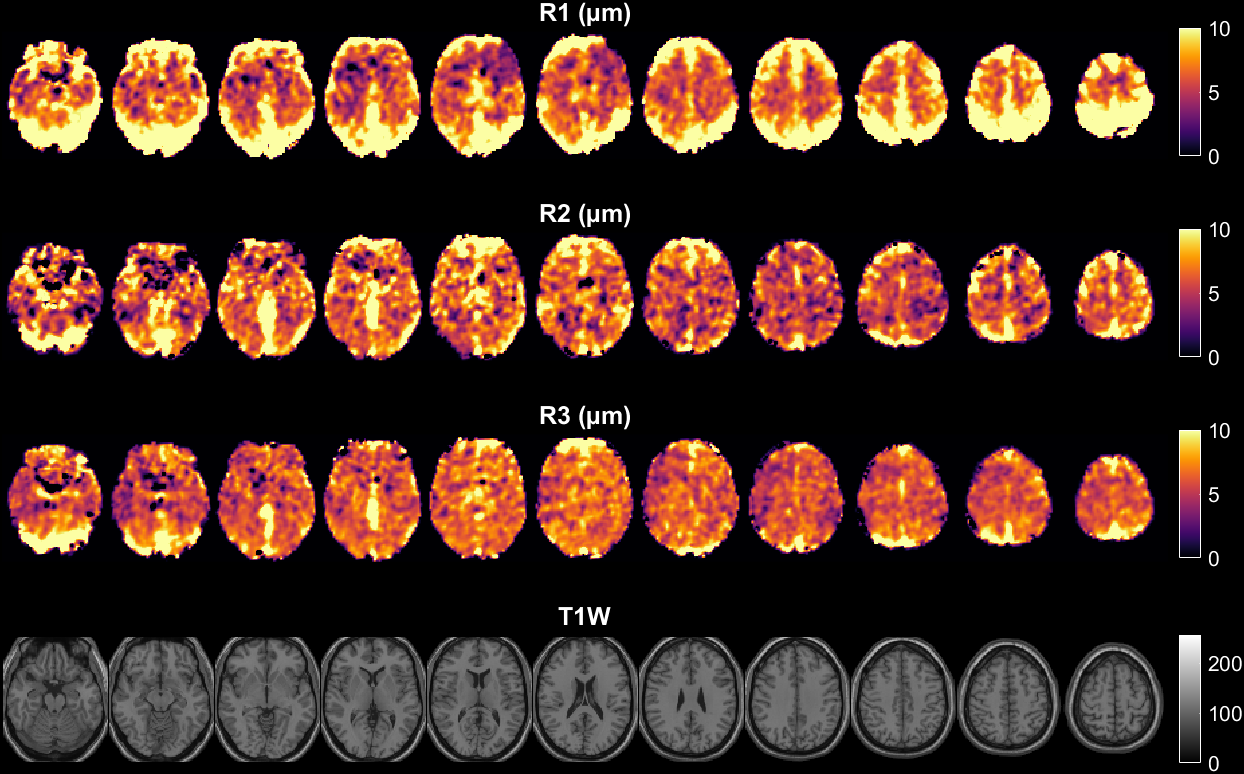


Figure S5. Calculated and spatially normalized vessel radius of the third represent subject (male, 28 years) after task (a) in day 1 with the title of R1, after task (a) in day 2 with the title of R2 and after task (b) in day2 with the title of R3.


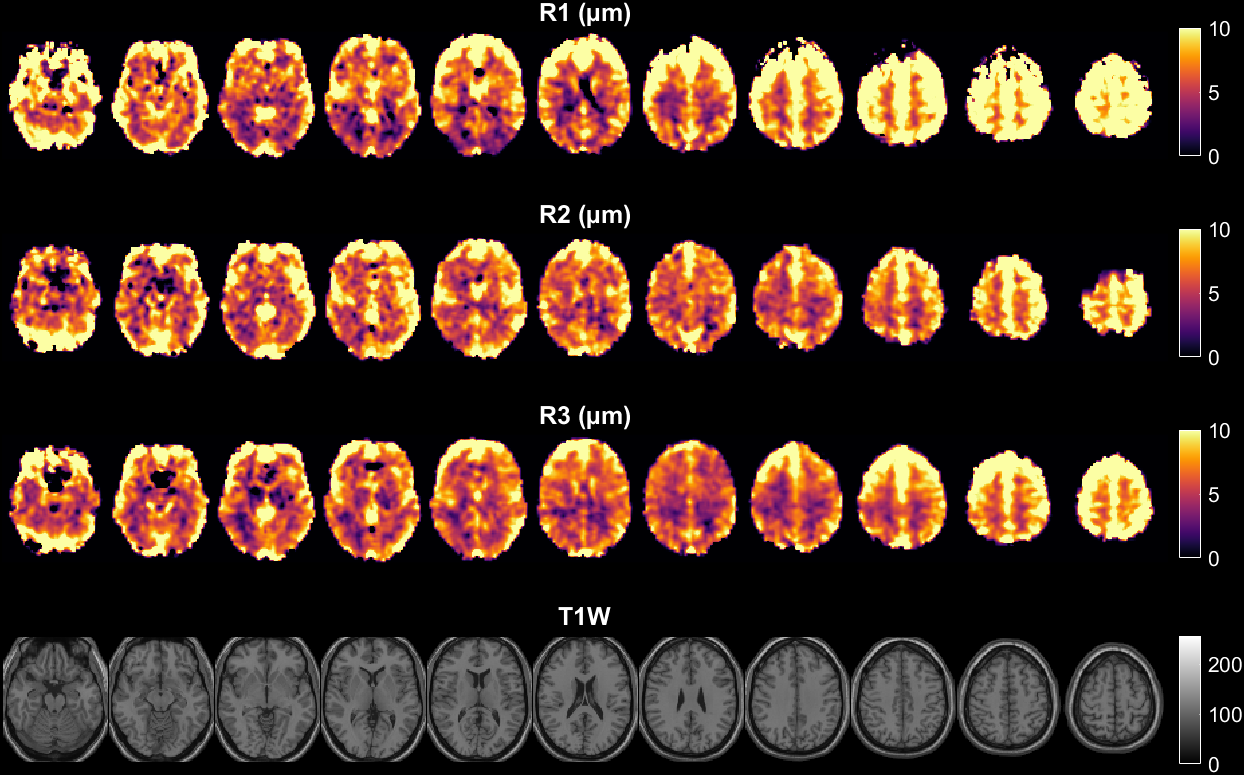


Table S1. Intra-class correlation coefficients (ICC) were calculate at five lobes of BH-VSI across three tests (R1-3) and across two paradigm at the same day (R2-3).

|  | ICC across all three tests | ICC across two paradigm at the same day |
| --- | --- | --- |
| Sub1 | 0.657 | 0.417 |
| Sub2 | 0.077 | 0.342 |
| Sub3 | 0.285 | 0.292 |
| mean±sd | 0.346±0.304 | 0.35±0.063 |
